# Phase Transitions in Boron Carbide

**DOI:** 10.3390/ma16206734

**Published:** 2023-10-17

**Authors:** Helmut Werheit

**Affiliations:** Faculty of Physics, University Duisburg-Essen, D-47048 Duisburg, Germany; helmut.werheit@koeln.de

**Keywords:** boron carbide, phase transition, structural disorder, electronic properties, phonons

## Abstract

The idealized rhombohedral unit cell of boron carbide is formed by a 12-atom icosahedron and a 3-atom linear chain. Phase transitions are second order and caused by the exchange of B and C sites or by vacancies in the structure. Nevertheless, the impact of such minimal structural changes on the properties can be significant. As the X-ray scattering cross sections of B and C isotopes are very similar, the capability of X-ray fine structure investigation is substantially restricted. Phonon spectroscopy helps close this gap as the frequency and strength of phonons sensitively depend on the bonding force and mass of the vibrating atoms concerned. Phase transitions known to date have been identified due to significant changes of properties: (1) The phase transition near the chemical composition B_8_C by clear change of the electronic structure; (2) the endothermic temperature-dependent phase transition at 712 K according to the change of specific heat; (3) the high-pressure phase transition at 33.2 GPa by the drastic change of optical appearance from opacity to transparency. These phase transitions affect IR- and Raman-active phonons and other solid-state properties. The phase transitions at B_~8_C and 712 K mean that a well-defined distorted structure is converted into another one. In the high-pressure phase transition, an apparently well-defined distorted structure changes into a highly ordered one. In all these cases, the distribution of polar C atoms in the icosahedra plays a crucial role.

## 1. Introduction

Boron carbide is one of the technically most important boron-rich solids. Outstanding properties such as low density (2.52 g/cm^3^), high melting point (T_m_~2700 K), and extreme hardness (~40,000 MPa) predestine boron carbide for application under conditions that are inaccessible for most other materials. Therefore, it is one of the best-investigated boron-rich solids where its structures are based on B_12_ or related icosahedra. The complexity of the structure and extraordinary electronic properties have attracted attention. Regarding technical application, boron carbide is the most important boron-rich material. Over the last century, its hardness and chemical stability have been utilized in powders for grinding and polishing, in nozzles for sandblasting, and in mortars (see A. Lipp [1]). Meanwhile, high-temperature ceramics and lightweight armoring of objects and persons have become important. High neutron absorption of the ^10^B isotope allows application for neutron shielding and control rods in nuclear devices. Domnich et al. [2] reviewed such applications. In general, due to its extraordinary properties, boron carbide is highly promising for application under extreme conditions, such as high temperatures and mechanical or chemical burdens. Mostly in such applications, details of the complex crystalline structure of boron carbide, such as the distribution of C atoms, are of subordinate significance.

This does not apply in the case of the electronic properties. Optical appearance and electronic transport properties characterize boron carbide clearly as a p-type semiconductor (see [3,4]). However, untypical behavior indicates the strong impact of structural defects.

The high Seebeck coefficient (S~300 mVK^−1^ up to at least 2000 K) is a promising potential for direct thermoelectric energy conversion (see [5]).

Further uncommon effects, such as low-T specific heat anomaly [6] and the effects of fast neutron irradiation on the thermal conductivity [7], might also be associated with structural changes.

At least concerning details, the crystalline structure of boron carbide has not yet been finally determined. The formerly frequently and even currently occasionally assumed representative compound B_4_C (structure formula (B_11_C) CBC) does not exist in reality. B_4.3_C is the real compound achieved in commercially produced boron carbide [8,9] and in single crystals [10]. If B_4_C is obtained, excess carbon is precipitated in the structure, typically in the form of graphitic layers [11,12]. Unlike B_4.3_C at the carbon-rich limit, the boron-rich limit of the homogeneity range is less well-defined. A few samples, whose C content was clearly below B_11_C, were unambiguously characterized as boron carbide, albeit with a large error margin of lattice parameters (see [13] and references cited therein). Otherwise, β-rhombohedral boron with clearly higher C content has been obtained (see [14] and references cited therein). Therefore, the coexistence of both structures in a certain range cannot be excluded. Theoretical studies claim the structure model (B_12_) CBC to be the energetically most favorable, while various experiments have proved this compound to be the most distorted one in the whole homogeneity range (see Reference [3]).

Phase transitions, structural changes related, and their effect on some other solid-state properties are the object of this paper.

## 2. Structure

**Figure 1:**
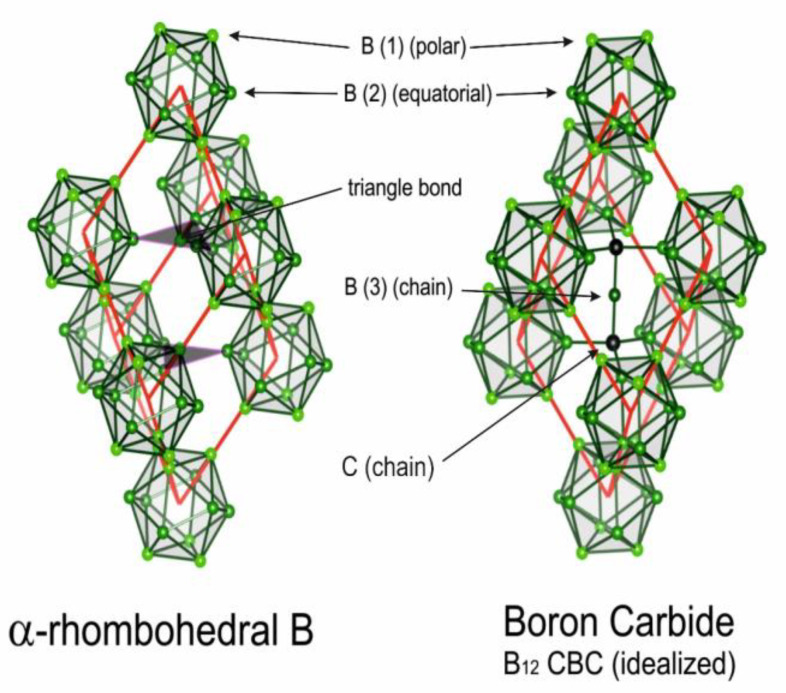
Idealized rhombohedral unit cells of α-rhombohedral (structure formula B_12_) boron and boron carbide (structure formula B_12_ CBC) [12]. In (B_11_C) CBC at the carbon-rich limit, the icosahedral C atom occupies one of the B(1) polar sites. Reprinted from Ref. [13] published by Elsevier Masson SAS. All rights reserved.

Figure 1 [13] shows the rhombohedral unit cells of α-rhombohedral (α-rh. B; idealized structure formula B_12_) boron and boron carbide (idealized structure formula (B_12_) CBC and (B_11_C) CBC), respectively. The actual structures differ more or less decisively—in α-rhombohedral boron, IR phonon spectra suggest the existence of additional single B atoms [15]. In boron carbide, where its homogeneity range extends from B_4.3_C at the carbon-rich to about B_11_C at the boron-rich limit, high-density structural defects have been identified and impact the electronic properties decisively [16,17].

The defects in boron carbide are mainly formed by the exchange of B and C atoms on regular sites or of minor concentrations on unoccupied sites. X-ray diffraction averaging larger volumes yields the basic parameters of boron carbide reliably, but it is largely unsuitable to analyze individual compositions of the components. Due to the similar X-ray scattering cross sections of ^10^B, ^11^B, and ^12^C isotopes, the capability of X-ray fine structure investigation is significantly restricted. Phonon spectroscopy helps close this gap.

**Figure 2:**
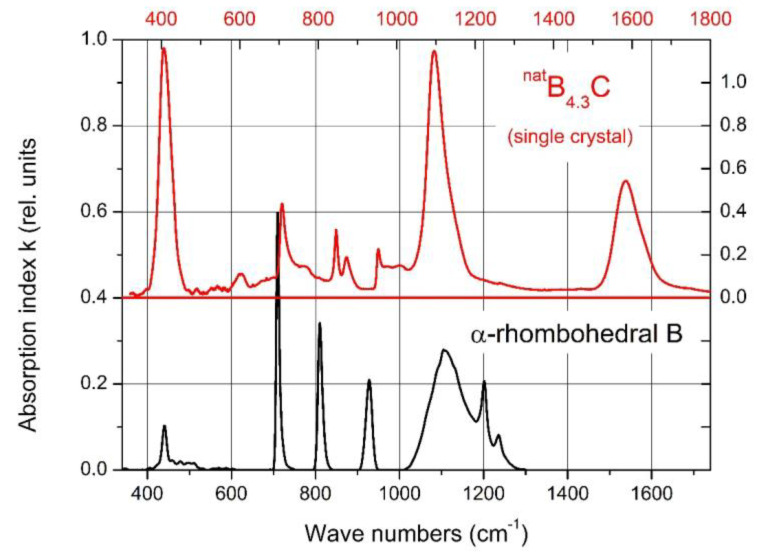
IR-phonon spectra of B_4.3_C boron carbide and α-rhombohedral boron. The abscissas are shifted relative to one another to facilitate the correlation of icosahedral vibrations.

**Figure 3:**
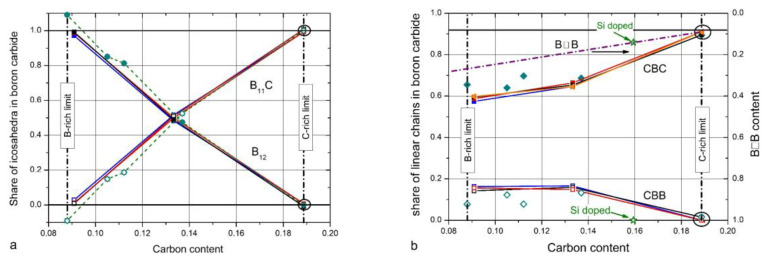
Shares of structure elements in the unit cell of boron carbide [16,22]; (**a**), B_12_ and B_11_C icosahedra, (**b**), CBC, CBB, and B☐B chains (☐, vacancy). Symbols connected with solid lines, isotope-enriched boron carbide; other symbols, polycrystalline boron carbide. Reprinted from Ref. [22] “© IOP Publishing. Reproduced with permission. All rights reserved”.

Helpful in this respect is the comparison of the spectra of IR- and Raman-active phonons with those of α-rhombohedral boron having the same icosahedral structure; however, without C atoms on polar sites of the icosahedron and without CBC or these replacing other structure elements. Figure 2 compares the IR-phonon spectra of single crystal ^nat^B_4.3_C boron carbide and α-rhombohedral boron (for comparison of the Raman spectra, see References [18,19,20]). The spectrum of α-rhombohedral B was discussed in Reference [15]. A detailed theoretical description was performed by Beckel et al. [21]. The spectra of boron carbide were analyzed by Werheit et al. (see Refs. [13,22] and the references cited therein). The concentration of structure elements depending on the C content is shown in Figure 3. Changes in some icosahedral vibrations in boron carbide in connection with phase transitions are discussed below.

The existence of the following defects in the structure in the form of deviations from the idealized structure formulas is generally confirmed:

- C atom replacing B(1) in (B_11_C) CBC; that means one of the six B atoms on polar sites of the icosahedra is replaced by a C atom. The distribution of these C atoms is an object of current investigation.
- Carbon-rich limit of the homogeneity range: B_4.3_C (instead of B_4_C, formerly assumed to be the actual compound of boron carbide) [6,7,14]. Hence, the real structure contains 5.7 at. % structural defects compared with the idealized (B_11_C) CBC structure. This also applies to high-quality single crystals, whose preparation is only possible in the chemical composition B_4.3_C [8], and to single crystals growing accidentally in industrial production (concluded from the electrical conductivity, see below Ref. [23]).
- Vacancies of the B(3) site (9–28%, depending on composition) (see [14] and references cited therein).
- CBB chains replace CBC with boron-rich boron carbides (Figure 3b, see [14] and references cited therein).

B_4.3_C is the least distorted compound [14,15]. It does not contain any free carbon, for example, systematically checked by Schwetz et al. [8,24] using a wet-chemical analysis for determining free and bound carbon in boron carbide separately. B_4.3_C is the only compound allowing the preparation of high-quality single crystals [8].

Theoretical model calculations determined the idealized structure formula (B_12_) CBC of the compound B_13_C_2_ as the energetically most favorable [25,26]. In fact, B_13_C_2_ is the most distorted boron carbide. Its highly complex structure (B_12_)_0.5_(B_11_C)_0.5_•(CBC)_0.65_(CBB)_0.16_(BB)_0.19_ shows that about 80% of the unit cells contain any structural defect [4,27,28]. High concentrations of structural defects (with regard to idealized structures) are a generic property of boron carbide structure.

The shares of structure elements in the unit cell depend on carbon content (see Figure 3 [22]), preferably from the investigation of IR- and Raman-active phonons. The comparably low share of B☐B chains (☐, vacancy) has not been measured but is used to close the gap between the sum of atoms in the other structure elements determined and the chemical composition. TEM studies by Rasim et al. [29] suggest that a variety of further kinds of structural elements can replace the CBC chain. However, apart from this TEM study, there is no further experimental evidence. Therefore, it cannot be excluded that such structural elements are provoked by the preparation process of the sample.

Many theoretical studies on structural details in boron carbide are based on Raman studies, which are affected by a severe systematic experimental error [18]. In cases of high Raman excitation energy, the spectra do not reflect bulk properties. Hence, the results of accordingly performed calculations merely end in themselves and do not yield scientific progress (see, for example, the controversial discussions in [19,30,31,32,33,34]).

## 3. Electronic Structure of B_4.3_C at Ambient Conditions

**Figure 4:**
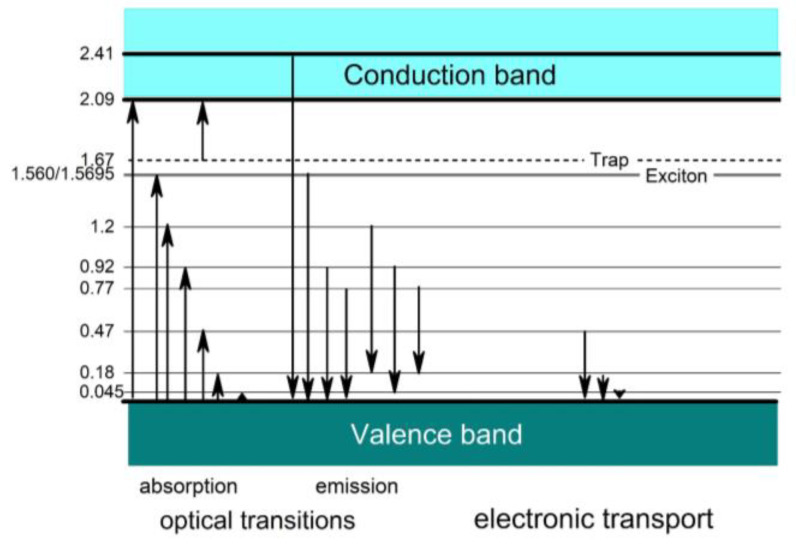
Empirical band scheme of boron carbide based on experimental results (see [3,4] and references cited therein).

**Figure 5:**
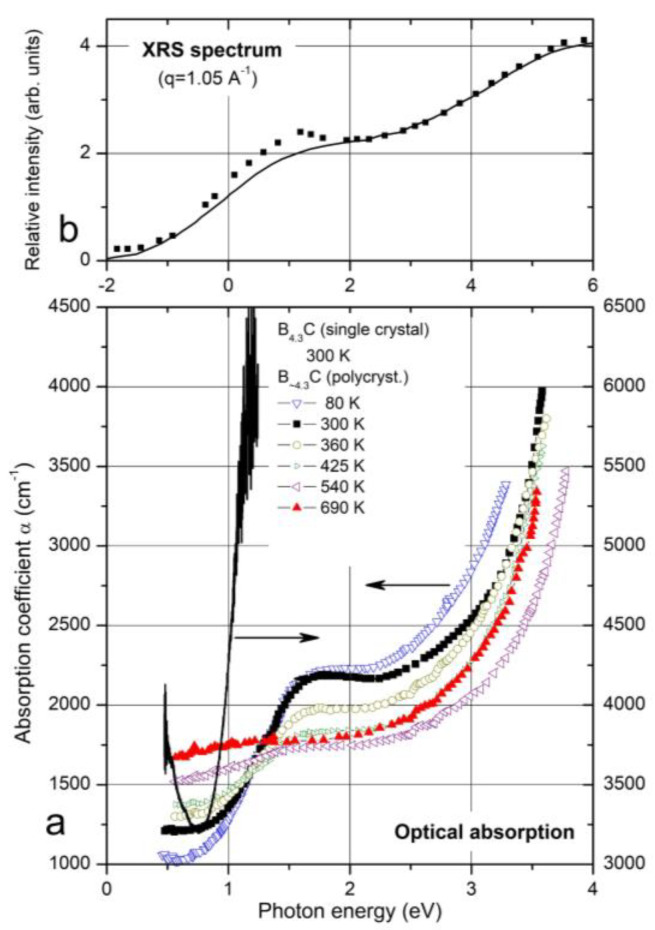
B_4.3_C. (**a**) Optical absorption edge [3,4,42]. Symbols, polycrystalline material; line, single crystal. (**b**) X-ray Raman scattering (XRS) spectrum [43]. Symbols selected experimental data for *q* = 1.05 °A^−1^; line, site-specific ab initio calculation for the background of icosahedral B atoms. We assume that the composition of this boron carbide corresponds to B_4.3_C, the carbon-rich limit of the homogeneity range; the composition B_4_C claimed by the authors does not exist (see above).

Due to the availability of material, previous experimental studies of electronic properties are mainly restricted to polycrystalline boron carbide and especially to the compound B_4.3_C. This also applies when the compound is nominally designed as B_4_C, as this is, in reality, B_4.3_C with excess carbon typically distributed in the form of graphitic layers [11,24]. High-quality B_4.3_C single crystals were used in References [10,35,36]. Figure 4 shows a collection of transition energies, consistently obtained from optical absorption such as those shown in Figure 5, indicating interband transitions at 2.09 and 2.41 eV and from electrical transport measurements (see [37,38,39,40,41] and references cited therein).

**Figure 6:**
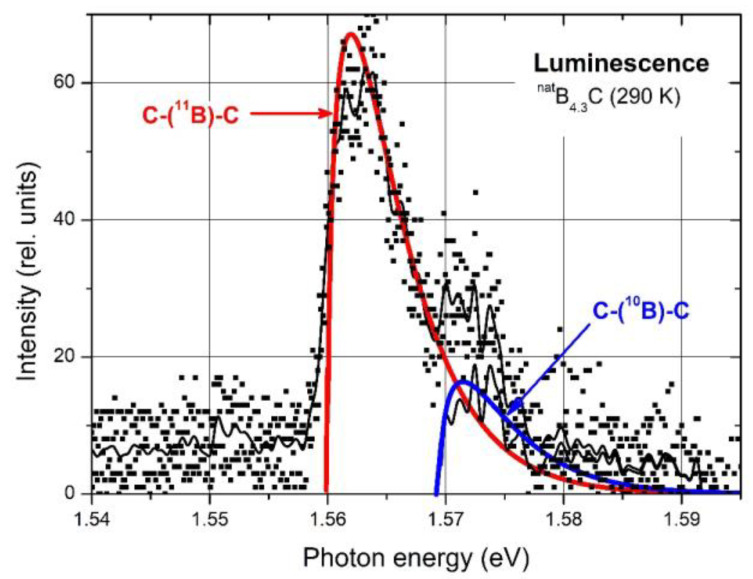
B_4.3_C, photoluminescence spectrum at 290 K [42]. Excitation with the 514.5 nm (2.4 eV) line of an Ar laser; intensity 280 mW mm^−2^. Squares, experimental results; thin black lines, averaged experimental results, before and after subtracting the 1.56 eV model fit, respectively; thick colored lines, recombination models of free excitons (1.560 and 1.5695 eV, respectively).

Off the absorption edge in Figure 5, there is a peak absorption in polycrystalline boron carbide at 1.56 eV. On the first view, surprisingly, the absorption of high-quality single-crystal boron carbide in this range is considerably higher. XRS emission (Figure 5, Feng et al. [43]) and luminescence [10,42] (Figure 6) suggest a p-type exciton associated with the central B atom of the CBC chain. The luminescence peak consists of two components, where the intensity relation is 4:1. This corresponds quite well with the relation in ^nat^B_4.3_C ^11^B/^10^B = 4.03 if random isotope distribution is assumed. The formation of excitons requires largely undistorted structures. In B_4.3_C single crystals, this condition is better fulfilled than in polycrystals, and at low T, it is better than at high T. This explains the peculiarities in Figure 5. Hence, the gap state “Exciton” in Figure 4 is not evoked by structural defects. However, the effects of structural defects on the optical absorption cannot be excluded [44]. For example, at high pressure (see below), structural distortion occurs by buckling out of the central B atom in the CBC chain [15,23,35,36].

Former theoretical studies on boron carbide were not very helpful in understanding the complex interactions between structural details and electronic properties [45,46,47,48,49,50,51], as they were based on idealized structure models deviating more or less considerably from the real structures. Thus, related conclusions were often in significant contrast to experimental results. For example, the theoretically claimed metallic character or even superconductivity [48] was opposite to the semiconducting behavior experimentally proved from the very beginning. Surprisingly, even in recent studies, the real structure of boron carbide is frequently ignored.

## 4. Phase Transition Near the Compound B_8_C

Reliable information on the electronic structure of more boron-rich boron carbide is obtained from electrical conductivity, measured between 5 and 2100 K by Werheit et al. [37], by Wood [38,39] and by Aselage et al. [40]. Results obtained by Amulele et al. [41] up to 9 GPa pressure are compatible.

**Figure 7:**
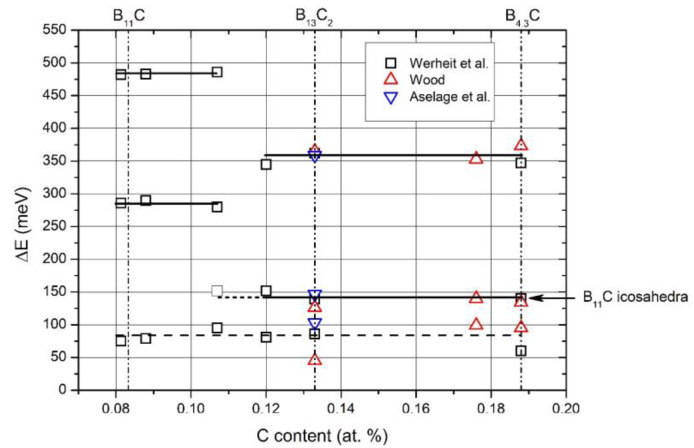
Phase transition near the compound B_8_C.

The activation energies obtained from the electrical conductivity are displayed in Figure 7, showing that at ~B_8_C, the homogeneity range is divided into two parts with significantly different electronic characteristics. The activation energy at about 80 meV found in both regions is uncertain as obtained in that range of temperature, where a continuous transition from thermal excitation to variable-range hopping takes place (see [37]).

**Figure 8:**
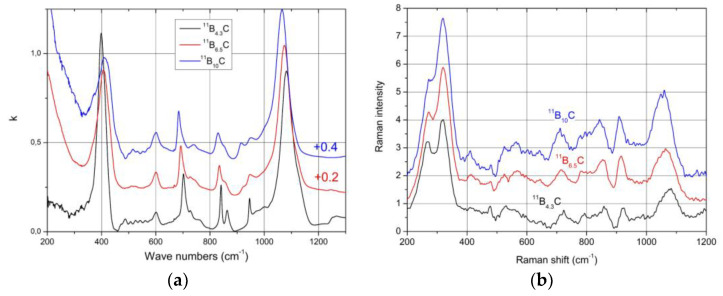
Phonon spectra of ^11^B_x_C at 30K [33,40]. (**a**), IR-active phonons; (**b**), Raman-active phonons.

It is expected that this phase transition is correlated with a structural change. This should be reflected in the phonon spectra. Figure 8 shows the IR- and Raman-active phonons of ^11^B_x_C boron carbide [52,53]. We analyze some phonons showing changes depending on the chemical composition.

On the basis of specific phonons, we check below how far structural changes are involved in the phase transitions. In most cases, the phonons concerned are no single modes but overlap in the spectrum. For separating the components, we used the “OriginPro software for graphic and analysis,” allowing us to fit overlapping Gauss-like or Lorentz-like resonances. The phonons, which are affected by passing the phase transition, cannot be precisely attributed to concrete atoms or atomic arrangement. Accordingly, model calculations, which could help clear this deficiency, are missing so far.

### 4.1. IR-Active Phonon Near 400 cm^−1^

**Figure 9:**
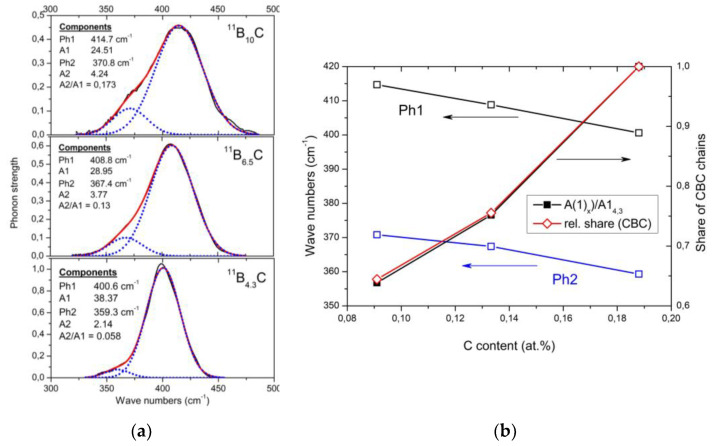
(**a**). IR-active phonon of ^11^B_x_C near 400 cm^−1^ (All fits were obtained using the Origin Pro software). (**b**). Phonon shift depending on C content and comparison between the reduction of phonon strength (components 1 and 2) and relation of the phonon strength of component #1 depending on C content compared with the relative share of CBC chains in the structure (obtained from the IR-active stretching vibration near 1600 cm^−1^ in Figure 2b).

The phonon near 400 cm^−1^ is composed of two components (see Figure 9a), where the frequencies (Figure 9b) and phonon strengths vary depending on the C content. Shirai et al. [54,55] and Vast et al. [50,56] attribute the IR phonon mode near 400 cm^−1^ to the central B atom in the chain vibrating perpendicularly to the chain axis [27]. Experimentally, this is confirmed for the component Ph 1, whose strength, depending on the C content, precisely matches the share of CBC chains (Figure 9b). The considerably weaker component Ph 2 of this phonon belongs to the icosahedral structure, accordingly with its counterpart in the spectrum of α-rhombohedral boron (see Figure 2). This phonon was already mentioned by Beckel et al. [21] but not described in detail (see [16]). Below, we perform an analysis of the temperature dependence of this phonon.

In Figure 9, no apparent change suggests a relation of this phonon to the phase transition at B_8.1_C. However, the mode Grüneisen parameter γ of the IR-phonon (−3.21 at low and +6.5 at high temperatures) deviating strikingly from all other phonons [36] suggests a peculiarity. The previously assumed involvement of this phonon in the T-dependent transition at 712 K [57] is checked below.

### 4.2. IR-Active Phonons Near 700 cm^−1^

**Figure 10:**
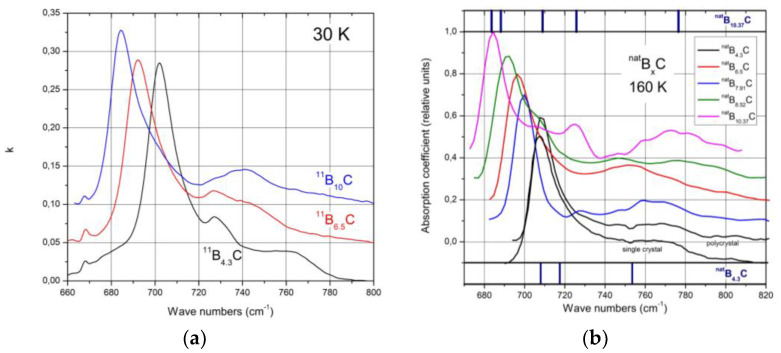
IR-active phonons near 700 cm^−1^. (**a**), ^11^B_x_C at 30 K; (**b**), ^nat^B_x_C at 160 K, phonon-wavenumbers of ^nat^B_4.3_C and ^nat^B_10.37_C.

**Figure 11:**
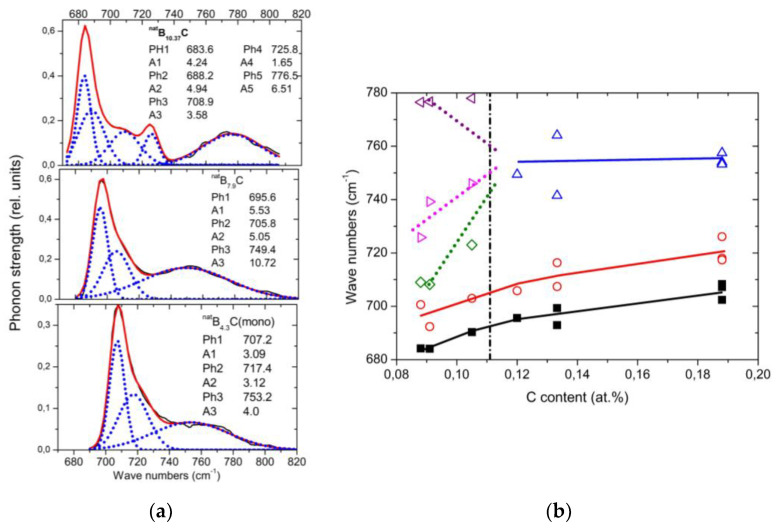
IR-active phonons near 700 cm^−1^. (**a**) Fits obtained with OriginPro software. (**b**) Wave numbers of phonon components vs. C content. The results of the high-frequency components of B_8.52_C (C content 0.105) are rather uncertain.

The spectra of isotope-enriched samples (Figure 10a) exhibit clear changes depending on C content. For interpretation, a denser sequence of spectra is required. Data on ^nat^B_x_C are available ([57] Figure 10b). The results of the analysis (examples in (Figure 11a)) are displayed in Figure 11b. Splitting of the component of this phonon near 760 cm^−1^ into three branches indicates a structural change at the phase transition. This phonon is correlated with the strong 705 cm^−1^ intra-icosahedral single-line A_2u_ mode in α-rhombohedral boron, described by Beckel et al. [21], split in the boron carbide structure (see [16,31]).

### 4.3. IR-Active Phonons Near 940 cm^−1^

**Figure 12:**
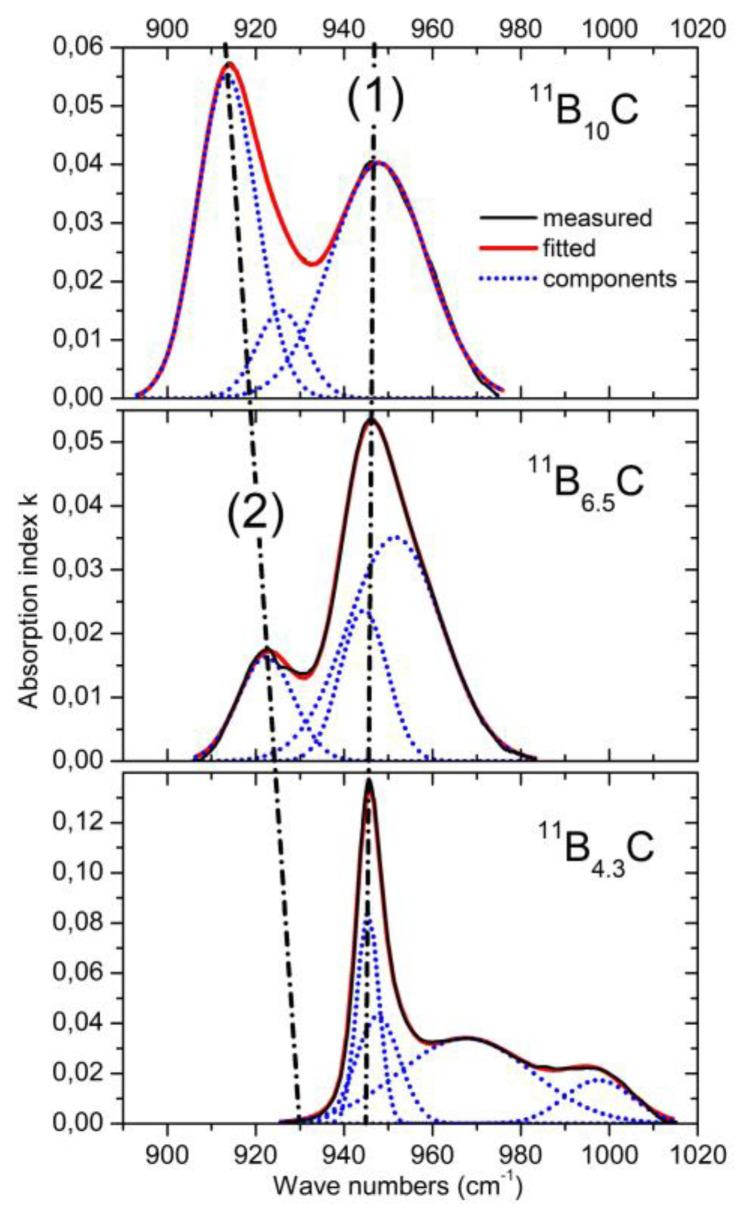
IR-active phonons near 950 cm^−1^. (1) and (2) mark the apparent shift and strength of specific components.

The phonon near 940 cm^−1^ (Figure 12) represents an icosahedral vibration proved by the counterpart in the spectrum of α-rhombohedral boron (see Figure 2). According to Beckel et al. [21], this phonon consists of superimposing E_u_ and A_2u_ modes. In boron carbide, the additional effect of polar C atoms in the icosahedra must be considered. This explains, in principle, the complexity of the phonon and of its change depending on C content.

However, there is a significant change between B_4.3_C and B_6.5_C: two components of the phonon vanish completely. Unfortunately, there are no spectra in the intermediate range available, which could help explain this behavior.

### 4.4. FIR-Absorption

**Figure 13:**
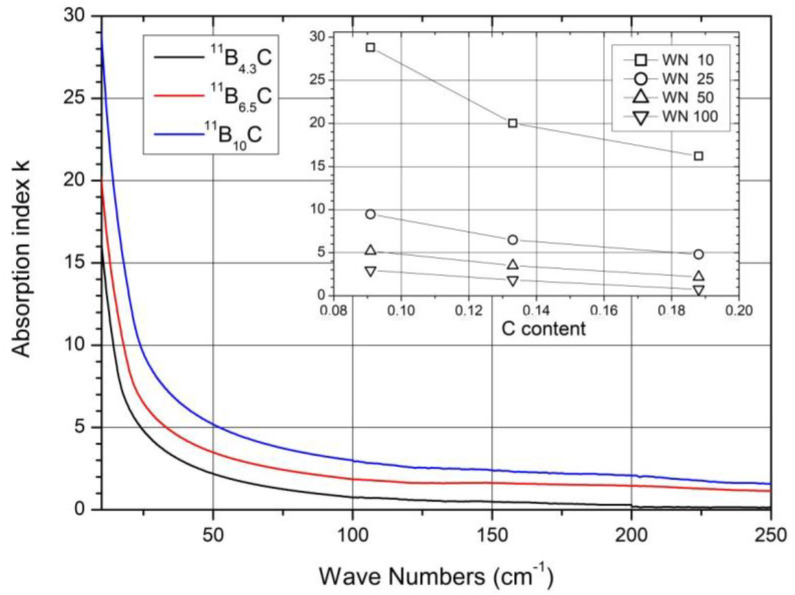
Heavily damped plasma absorption in ^11^B_x_C at 30 K [28].

As indicated in the low-frequency region of Figure 8a, the FIR optical absorption increases towards very low wave numbers. Figure 13 shows this range extended down to 10 cm^−1^. This absorption is caused by the plasma-like absorption of free charge carriers, which is heavily damped in boron carbide (for details, see [28]). With respect to the electric structure, Figure 13 shows that the concentration of free carriers increases with the C content decreasing. However, there is no effect of the phase transition at B_~8_C on the concentration of free holes discernable.

The share of B_11_C icosahedra decreases from ~1 in B_4.3_C to ~0.1 in B_10_C (see Figure 3). The main components (1) and (2) seem to vary continuously depending on the C content. There is no indication of a sudden change near B_8_C. Therefore, no involvement of this phonon in the phase transition at B_~8_C is discernible.

### 4.5. Raman-Active Phonons

**Figure 14:**
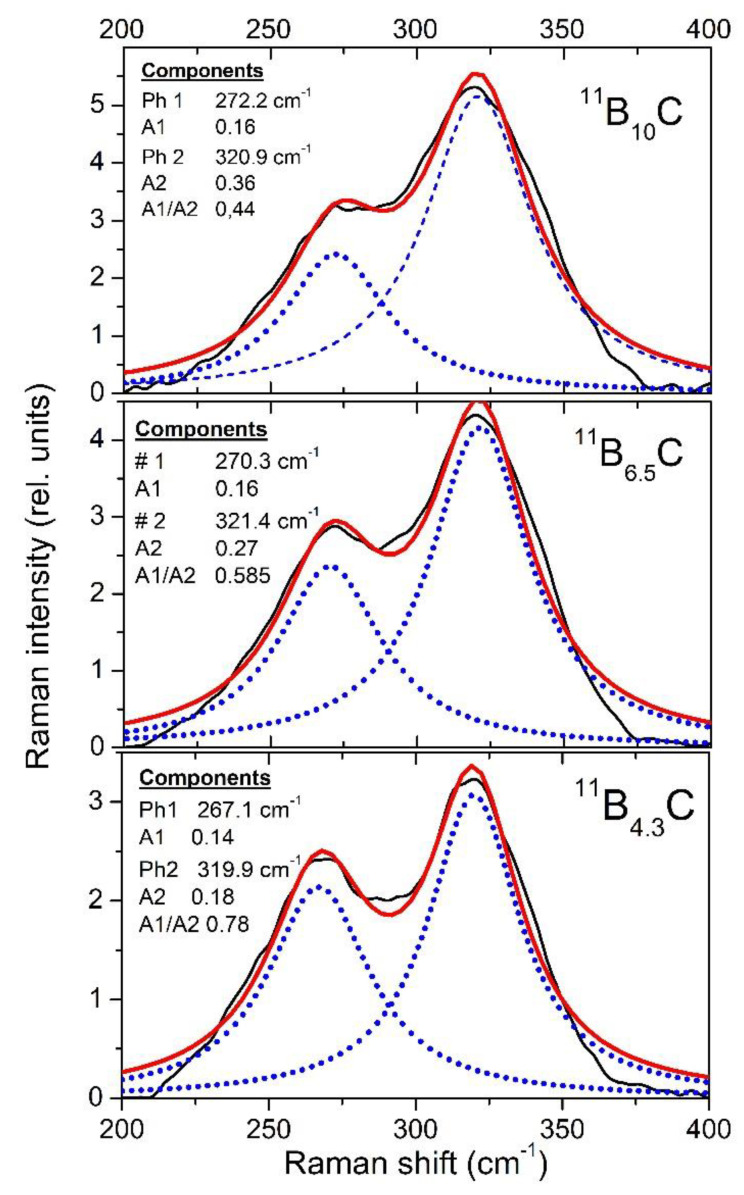
Raman-active phonons of ^11^B_x_C at 270/320 cm^−1^.

The resolution of the Raman spectra in Figure 8b is restricted. Therefore, variations dependence on the C content cannot be doubtlessly identified, apart from the strong phonon doublet at 270/320 cm^−1^ (Figure 14). This has no counterpart in the spectrum of α-rhombohedral boron. Therefore, it is to be attributed to the structural changes in boron carbide by C atoms: B_11_C icosahedra, CBC, or CBB chains.

We attribute this doublet to the E_g_ phonon at 335 cm^−1^ described in the theoretical study by Shirai and Emura [55] as a rotation of the CBC chain associated with a wagging icosahedron and assume that the splitting could result from the occupation of the atomic sites involved partially occupied by C or B atoms (see [12]).

The strengths of both components change monotonically depending on the carbon content. There is no indication that this phonon is involved in the phase transition at B_~8_C. 

## 5. Phase Transition at 712 K

**Figure 15:**
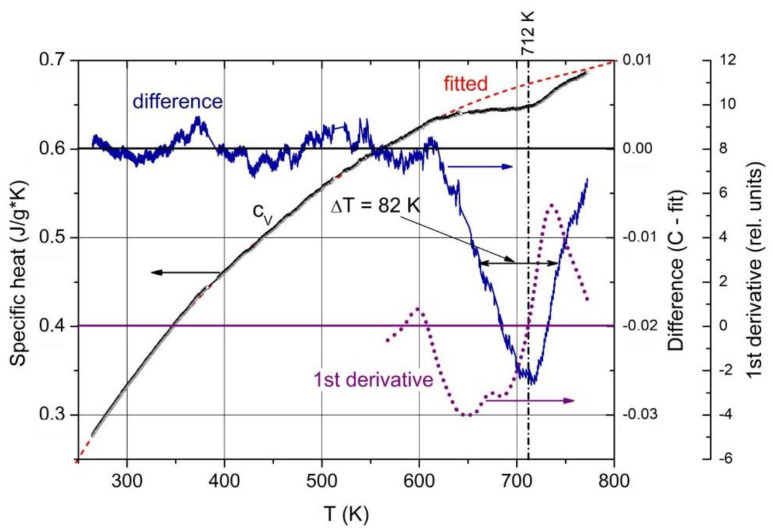
Specific heat of B_4.3_C boron carbide vs. T. Black circles, measured; red dashed line, polynomial fit; blue, difference between measured data and fitted line; purple dotted line, 1st derivative.

The T-dependent specific heat (Figure 15) shows an exothermic phase transition at 712 K, half-width 82 K [58]. The electrical conductivity proves the correlation of this phase transition with a structural change, where the activation energy of 140 meV is correlated with the content of the B_11_C icosahedra and polar C atoms, respectively (see above). At carbon contents below the phase transition B_~8_C, it is no longer detectable [50].

Yao et al. [59] described theoretically in this range an endothermic phase transition that is related to the site exchange of carbon atoms within the icosahedra. Obviously, this process is not compatible.

**Figure 16:**
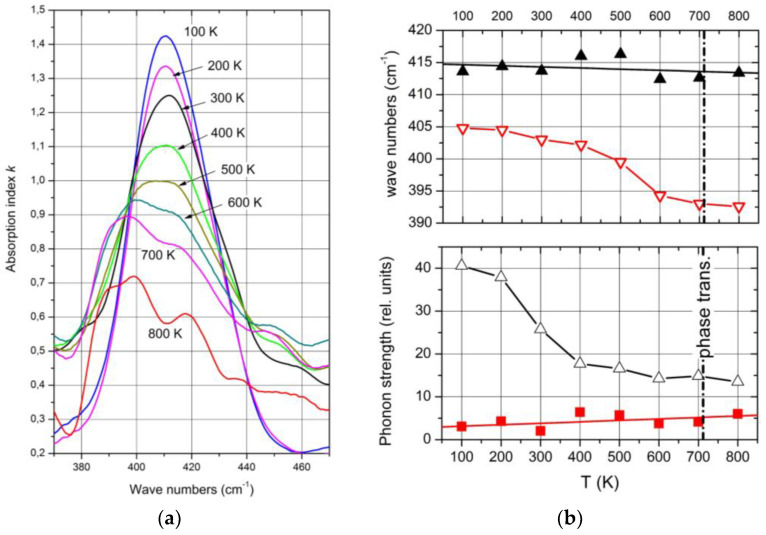
IR-active phonon of ^nat^B_4.3_C boron carbide near 400 cm^−1^. (**a**) Absorption index k vs. wave number between 100 and 800 K; (**b**) wave number and phonon strength of the components vs. T. Black, bending mode of the CBC chain; red, icosahedral vibration.

Complete temperature-dependent phonon spectra of boron carbide are available below 450 K only. The exception is the IR-active phonon mode near 400 cm^−1^, available between 100 and 800 K (Figure 16a) [28]. The spectra suggested a structural change by mode splitting near the phase transition. However, this splitting is feigned.

As shown above, the IR-active phonon mode near 400 cm^−1^ consists of two components. One of them is an icosahedral vibration identified by the equivalent vibration in α-rhombohedral boron (see Figure 2). As such, it is mentioned by Beckel et al. [21] but not described in detail. Shirai et al. [54,55] and Vast et al. [51,56] attribute the second component to the central B atom in the CBC chain vibrating perpendicularly to the chain axis. We analyzed the IR absorption of this phonon accordingly. The parameters of both components are displayed in Figure 16b, showing that the before assumed split was feigned by the considerably different temperature dependence of the components.

Passing the phase transition, the frequency of the component representing the icosahedral vibration decreases clearly from 405 cm^−1^ at low T to 395 cm^−1^. Simultaneously, the phonon strength concerned increases marginally, showing that the number of vibrating elements remains nearly unchanged. The atomic bonding force concerned falls slightly in the range of phase transition but is nevertheless clearly measurable. It is surprising that the range of decrease is wider than the half-width of the phase transition (Figure 14). A certain effect of the phase transition on the electronic properties is clearly established.

The vibration of the central B atom in the chain behaves differently. The frequency remains nearly constant, showing that the bonds within the CBC chain are hardly affected by T. In contrast, the phonon strength representing the number of vibrating elements decreases considerably.

A certain difference between the structures below and above the phase transition is indicated by the frequency of the icosahedral phonon near 400 cm^−1^ (Figure 16b), which decreases from 405 cm^−1^ at low T to 392 cm^−1^ at high T. In contrast, the frequency of the bending mode of the central B atom in the CBC chain decreases at most marginally from ~414 to ~407 cm^−1^. The redistribution of polar C atoms in the phase transition affects the bonding force within the icosahedron and, consequently, the electronic structure as well.

An important further characteristic of this phase transition can be obtained from the electrical conductivity at pressures up to 9 GPa [41]. The phase transition is reversible, however, with considerable relaxation time.

## 6. Phase Transition at 32 GPa

**Figure 17:**
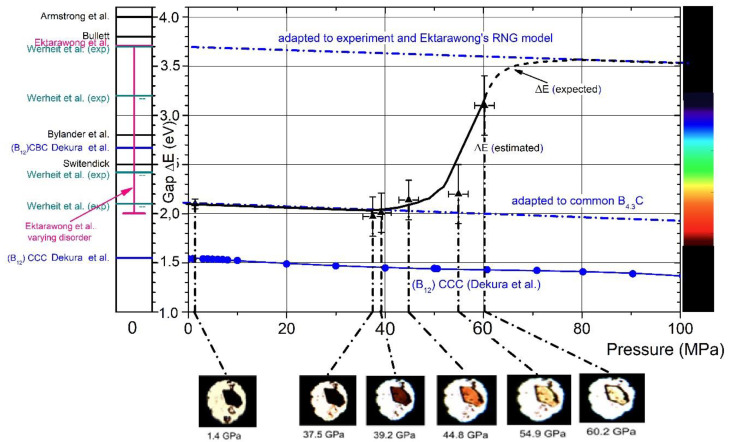
The gap width of boron carbide depending on pressure. Black triangles, gap width, roughly estimated from transient-light photos [35]. Compared with experimental and theoretical results [45,46,60,61]; pink, ordered structure [60]; pink vertical bar, varying configurational disorder [60]; blue filled circles, pressure-dependence [35]. Experimental results: cyan, ambient conditions [62,63]; dash-dotted lines, adapted to relevant gap widths at 0 GPa for distorted and undistorted B_4.3_C (Ektarawong’s RNG model), respectively; pressure-dependence according to the calculation for (B_12_)CCC [61].

The drastic change from opacity to glasslike transparency is the visual appearance of the phase transition in boron carbide at 32 GPa. Hushur et al. [35] used the photos shown in Figure 17 to estimate the band gap increasing at increasing pressure roughly. This implies that the electronic structure of boron carbide known from ambient conditions [15] (see Figure 3) changes fundamentally at high pressure.

In Figure 17 [35], the estimated shift of the band gap is compared with theoretical model calculations on undistorted and distorted boron carbides [45,46,60,61] and experimental results [44,62,63]. Obviously, this phase transition converts boron carbide, as it is commercially available or prepared at a laboratory scale and is highly distorted, into a largely undistorted state, which is at least close to the idealized models used in theoretical calculations.

At rising pressure, the band gap increases from values near 2 eV, experimentally determined [60] and theoretically attributed to a strongly distorted boron carbide [45] to gap widths between 3 and 4 eV, theoretically predicted for idealized meaning defect-free boron carbide.

Ektarawong et al. [45] studied how the distribution of icosahedral C atoms affects the electronic DOS. They used the idealized (B_11_C) CBC) structure and ignored changes in the CBC chain. In the icosahedra, six polar B(1) and six equatorial B(2) sites are occupied by B atoms, apart from one upper polar site p1 substituted by C. This C atom is slightly shifted towards the center of the icosahedron (see Reference [25] and references cited therein). The energetically most favorable defect structures are generated by the transfer of the C atom to both neighboring p2 and p3 sites, respectively, followed by transfers from p1 to the lower polar sites p4, p5, and p6, respectively. Ektarawong’s RNG model is based on the assumption that the polar C atoms are randomly distributed over all six polar sites.

Rasim et al. [29] take the idealized (B_12_) CBC structure model for reference. As mentioned above, this model is not compatible with real boron carbide. Nevertheless, this study excludes interfering effects in icosahedra when calculating the effects on the DOS by exchanging the CBC chain with other structure elements of various chain structures.

**Figure 18:**
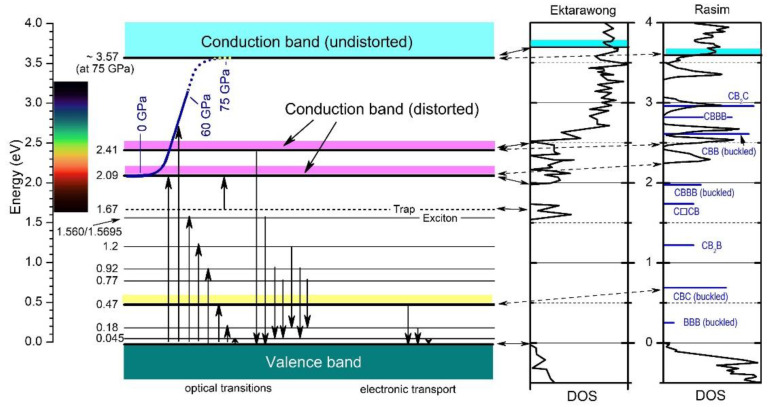
Electronic band scheme of B_4.3_C boron carbide [17] derived from optical and electrical measurements; DOS calculated by Ektarawong et al. (RNG model) [45] and Rasim et al. [29] for reference (data taken from diagram each). Vertical arrows show absorption (upward) and emission (downward) processes; the visible range is marked. Reprinted from Ref. [17] published by Elsevier Masson SAS. All rights reserved.

Both approaches mark significant achievements compared with the previous theoretical studies based on idealized and, therefore, unrealistic structure models. In Figure 18 [17], we check to what extent the DOS of both model calculations are compatible with the electronic transitions in the empirical band scheme (Figure 4) based on experimental results. These fit excellently with Ektarawong’s RNG model, assuming a random distribution of icosahedral C atoms over all the polar sites [45].

Considering these results, the strong optically excited transitions between ~2 and ~3.5 eV, which are responsible for the opacity of boron carbide at ambient pressure, are evoked in any way by the polar C atoms in boron carbide. The strong optical absorption concerned fulfills the requirements of electronic interband transitions and has been interpreted accordingly. Compared with classical semiconductors, the polar C atoms in boron carbide differ decisively from dopants having one excess electron compared with the basic lattice atoms and form low-density states in the gap. In boron carbide, the distribution of polar C atoms at room temperature induces high-density electronic states. A “simple” redistribution of the polar C atoms at high pressure, apparently to a higher degree of order, makes these high-density states completely disappear. This redistribution is reversible. That means the largely defect-free structure at high pressure [43] is metastable only, while the distorted structure is the thermodynamically stable one, at least under ambient conditions.

The reversibility of the structural distortion induced by the C atoms randomly distributed over all six polar sites stands in fundamental contrast to a sometimes-used model based on the assumption that the actual distorted structure of boron carbide is a non-equilibrium state only [28].

The DOS of boron carbide affected by structural elements replacing the CBC chain, calculated by Rasim et al. [29], is obviously not compatible with the high-pressure phase transition. Indeed, some experimentally determined narrow levels in the band scheme may correlate with specific structural elements assumed to replace the CBC chain. However, the related optical absorption is far too small to explain the strong absorption in this range at ambient conditions.

**Figure 19:**
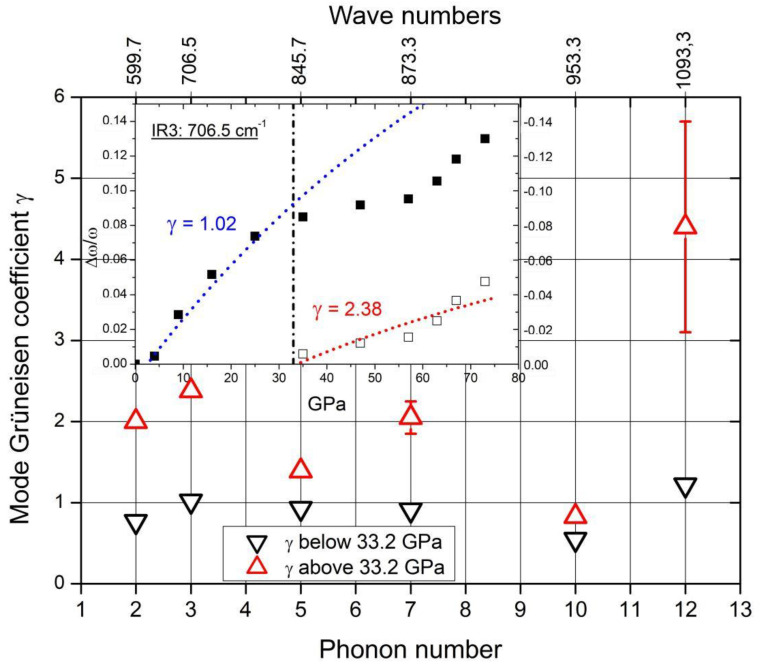
IR phonon mode Grüneisen parameters below (▽) and above (△) the phase transition at 33.2 GPa. Insert: an example of determining γ.

Therefore, the rearrangement of polar C atoms means a structural change and should be verifiable in the phonon spectra. The whole structure is affected by the phase transition at 33 GPa. This is proved by the phonon mode Grüneisen parameters γ. In the case of all IR- and Raman-active phonons, γ differs more or less considerably at pressure below and above the phase transition [13,30,36]. Unfortunately, in the pressure-dependent IR-phonon spectra of Chuvashova et al. [30], accordingly commented in [31] (some results are shown in Figure 19), the IR-active phonon near 400 cm^−1^ has been neglected. As shown above, a component of this phonon is sensitive regarding polar C atoms in B_11_C icosahedra.

The spectra of IR- and Raman-active phonons (Figure 8) exhibit strong, probably partially split peaks near 1100 cm^−1^. These are at least partially attributed to intra- and inter-icosahedral vibrations of polar atoms in the icosahedra. Hence, they should be affected by the rearrangement of polar C atoms.

In Figure 19, the Grüneisen coefficient of the 1093.3 cm^−1^ IR phonon above the phase transition considerably exceeds that of the other phonons. Moreover, the difference in the value below the phase transition is especially large. This suggests that the redistribution of polar C atoms increases the bonding force considerably.

**Figure 20:**
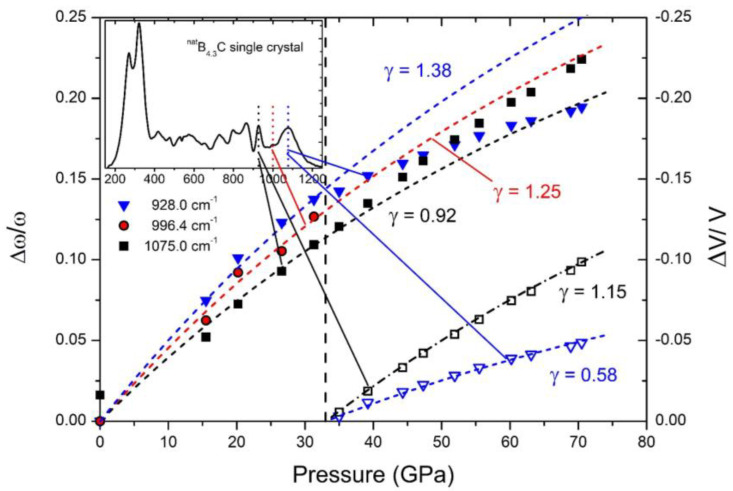
Relative shift of phonon frequencies (left ordinate) and unit cell volume (right ordinate) vs. pressure, determining mode γ of typical IR-active phonons of B_4.3_C boron carbide [13]. Insert: FT-Raman spectrum of single-crystal B_4.3_C boron carbide at ambient conditions. Reprinted from Ref. [13] published by Elsevier Masson SAS. All rights reserved.

The Grüneisen coefficients concerning Raman-active phonons, determined in Figure 20, change significantly at the phase transition due to rearranging polar C atoms.

In the Raman spectra of boron carbide, the phonon doublet at 270/320 cm^−1^ is the most striking feature. Werheit et al. [12] assigned this doublet to the 335 cm^−1^ *E*_g_ phonon that Shirai and Emura [55] attributed to a rotation of the 3-atom chain combined with a wagging icosahedron. Firstly, it is surprising that this phonon splits, though there are only B_11_C icosahedra in B_4.3_C. This could be explained as follows. The translation vectors of atoms involved in this phonon (Figure 1 in Reference [53]) show that 8 of 12 atoms in the icosahedra and 4 of 6 polar sites, respectively, are involved. Assuming that the C atom is randomly distributed over all polar sites of the icosahedra, there are B_11_C icosahedra with C atoms on sites that are involved in this vibration, while others contain exclusively B atoms on such sites. Due to different m_red_, the phonon mode splits and the components Ra 1 (~270 cm^−1^; C on one of the relevant sites) and Ra 2 (~320 cm^−1^; B atoms only) could be attributed accordingly.

The negative Grüneisen coefficients of both components differ fundamentally from all other phonons. Normally, compression reduces the lattice parameters, leading to a positive Grüneisen parameter, at least when the force constant is unaffected. However, that seems to be the case in boron carbide in connection with the rearrangement of polar C atoms at the high-pressure phase transition.

**Figure 21:**
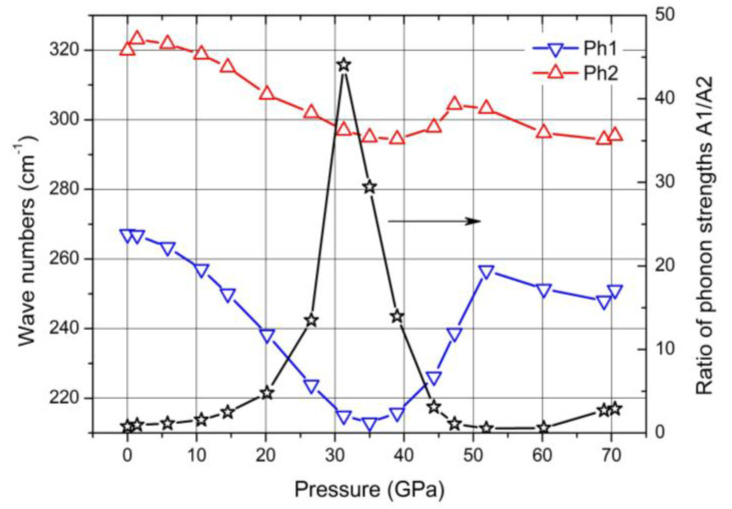
Wave numbers of the Raman-active 270/320 cm^−1^ doublet and ratio of the phonon strengths of both components.

In Figure 21, some parameters of both components of this doublet are displayed. For the evaluation, it had to be considered that the spectra were obtained using a laser with problematic excitation energy (for details, see [17]). While the phonon frequencies are not affected this way, the phonon strengths A1 and A2 derived from the measured spectra depend uncontrollably on pressure. This influence is eliminated by determining the ratio A1/A2 of both components shown in Figure 20. Based on the description of the 270/320 cm^−1^ phonon doublet above, Figure 21 indicates a strong site exchange of polar C atoms within the phase transition around 32 GPa.

## 7. Discussion

The electronic structure of boron carbide is dominated by B_11_C icosahedra. In comparison, the influence of the 3-atom chain is of minor importance. This is obvious in the phase transition near the chemical composition B_~8_C, which is related to the concentration of B_11_C icosahedra. The fact that this transition occurs at about 25% B_11_C instead of 50% shows the particular influence of the polar C atom that is missing in the frequently used hypothetical structure model B_12_CBC. Whether the distribution of one C atom on six possible polar sites has any effect within this composition-dependent phase transition remains open.

However, the distribution of polar C atoms is doubtlessly important in B_4.3_C with ~100% B_11_C icosahedra. The phase transitions at 712 K and 33.2 GPa are directly related to the distribution of the polar C atoms. At ambient conditions, boron carbide is opaque. Its optical absorption in the visible range of the optical spectrum is high due to optical transitions related to gap states shown in Figure 4. Hushur et al. [43,55] showed that high pressure changes the opacity drastically to transparency, indicating a highly ordered structure. However, this is metastable only, as it retransforms into the disordered state, reverting to normal conditions, albeit with considerable relaxation time. Accordingly, the well-known distorted structure is energetically favored at ambient conditions. Applying this knowledge to the hypothetical structure B_12_CBC possibly explains why this highly ordered structure is unstable compared with the distorted structure of natural boron carbide.

This leads to the conclusion that Ektarawong’s RNG model, assuming a random distribution of polar C atoms over all the polar sites at ambient conditions, is close to reality. The phase transitions at 712 K and 33.2 GPa both transform this distorted structure of boron carbide at ambient conditions (statistical distribution of 1 C atom per icosahedron to 6 polar sites) into a structure of higher order. This means the occupation of preferred polar sites; however, the actual distribution remains open. More detailed characterization of the phonon spectra might help.

In this study, the phonon spectra of boron carbides with natural isotope distribution and those highly enriched with ^11^B isotopes have been analyzed. Thus, isotopes could have a certain effect (for details, see [64]). The force constants of different isotopes are the same. Hence, the shift of the icosahedral phonon frequencies in B_4.3_C boron carbides, which are enriched with ^12^C and ^13^C, respectively (see spectra in [40,65]), yields the relation of the reduced masses involved. 

## Data Availability

Data are available on request.
